# Supplementary material for: Pain(less) cleansing: Watching other people in pain reduces guilt and sadness but not shame
Source: PLoS One. 2020 Dec 30;15(12):e0244429. doi: 10.1371/journal.pone.0244429 (PMC7773247; doi:10.1371/journal.pone.0244429)

**Videos used in all studies**

Pain Activation: <https://www.youtube.com/watch?v=1U1bbLxTFIA>

Excitement Activation: <https://www.youtube.com/watch?v=juVSJwUfbDc>

Control: <https://www.youtube.com/watch?v=GyZlJBnKoF0>

**Study 1**

In study 1, we additionally measured how painful the blood collection was according to the participants’ opinion and how much they identified with the protagonist. Participants were asked to report on 5-point scale their judgment from 1 = *not at all* to 4 = *extremely*. One-way ANOVA showed no differences between all three conditions for both pain perception, *F*(2, 57) = 1.38, *p* = .259, and identification with the actor, *F*(2, 57) = 1.03, *p* = .365.

**Additional analyses**

**Guilt.** The interaction between condition and time remained significant when we controlled for sadness both at Time 1 and Time 2, *F*(2, 55) = 5.25, *p* = .008, η_p_^2^ = .16, for shame *F*(2,55) = 5.71, *p* = .006, η_p_^2^ = .17 and when we controlled for the difference between positive and negative affect at Time 1 and Time 2, *F*(2, 55) = 6.10, *p* = .004, η_p_^2^= .18.

**Sadness**. The interaction between condition and time remained significant when we controlled for guilt both at Time 1 and Time 2, *F*(2 ,55) = 3.54, *p* = .036, η_p_^2^ = .14, but dropped to marginal significance when we controlled for the difference between positive and negative affect at Time 1 and Time 2, *F*(2, 55) = 2.94, *p* = .061, η_p_^2^ = .10

**Study 2**

**Pilot Study**

We chose movies used in Study 2 based on a pilot study in which 45 undergraduate students watched one of three videos: 1) the pain video from Study 1, 2) the bungee jump, or 3) the gnu movie and Afterwards rated how painful and exciting the scene was in the movie. A planned contrast analysis for one-way ANOVA (-1 gnu, 0 bungee jump, +1 pain) demonstrated that participants observed the pain movie as more painful (*M* = 2.73, *SD* = 1.94) than the gnu (*M* = 1.00, *SD* = .00) and bungee jump movies (*M* = 1.47, *SD* = 1.06), *t*(14) = 3.45, *p* = .004. The excitement results showed that the bungee jump movie was more exciting (*M* = 4.47, *SD* = 1.60) than the gnu (*M* = 1.40, *SD* = 0.63) and pain movies (*M* = 3.33, *SD* = 1.59), *t*(18,329) = 4.38, *p* < .001.

In study 2, we additionally measured how painful the blood collection was according to the participants’ opinion and how much they identified with the protagonist. Participants were asked to report on 5-point scale their judgment from 1 = *not at all* to 5 = *extremely*. One-way ANOVA showed no differences between all conditions for both pain perception, *F*(2, 150) = .32, *p* = .730, and identification with the actor, *F*(2, 150) = .67, *p* = .513.

**Full analyses**

**Guilt and sadness*.*** We analyzed the data using a mixed-design analysis of variance with a 2 (Condition: Guilt vs. Sadness) × 3 (Movie Activation: Pain vs. Arousal vs. Control) × 2 (Measured Emotion: Guilt vs. Sadness) x 2 (Time of the Measure: Time 1 vs. Time 2) design in which first two factors were between-group and the two remaining were within-group. The interaction of the highest degree was nonsignificant, *F*(2, 150) = 1.97, *p* = .375. However, we found a main effect of time, *F*(1, 150) = 12.26, *p* = .001, η_p_² = .08. Regardless of which emotion was measured, they were stronger at Time 1 (*M* = 3.14, *SE* = .10) than at Time 2 (*M* = 2.65, *SE* = .10). The main effect of the measured emotion was also significant, *F*(1, 150) = 78.07, *p* < .001, η_p_² = .34. Guilt was stronger than sadness at each time of measurement (*M* = 3.47, *SE* = .11 vs. *M* = 2.31, *SE* = .07).

The next significant effect was an interaction between condition and measured emotions, *F*(1, 150) = 52.01, *p* < .001, η_p_² = .26, which we described in the section about manipulation control in the article. The effect of movie activation was also significant *F*(2, 150) = 4.86, *p* = .009, η_p_^2^ = .06, which means that emotions in the pain activation movie (*M* = 2.63, *SE* = .12), were weaker than emotions in the control movie (*M* = 3.15, *SE* = .12, *p* = .006) but at the same level as emotions in the arousal activation movie (*M* = 2.90, *SE* = .12, *p* = .286). There was no difference between the last two conditions, *p* = .287. The effect of condition was also significant *F*(1, 150) = 6.24, *p* = .014, η_p_² = .04, which mean that emotions in the guilt condition were stronger than emotions in the sadness condition (*M* = 3.07, *SE* = .10 vs. *M* = 2.72, *SE* = .10).

The last significant effect was an interaction between condition, measured emotion and time of measure, *F*(1, 150) = 5.49, *p* = .020, η_p_^2^ = .04. As presented in Figure S1, this effect mainly shows that measured emotions decreased at Time 2 when they were activated (guilt in the guilt condition and sadness in the sadness condition), but they remained at the same level when they were measured but not activated (guilt in the sadness condition and sadness in the guilt condition).


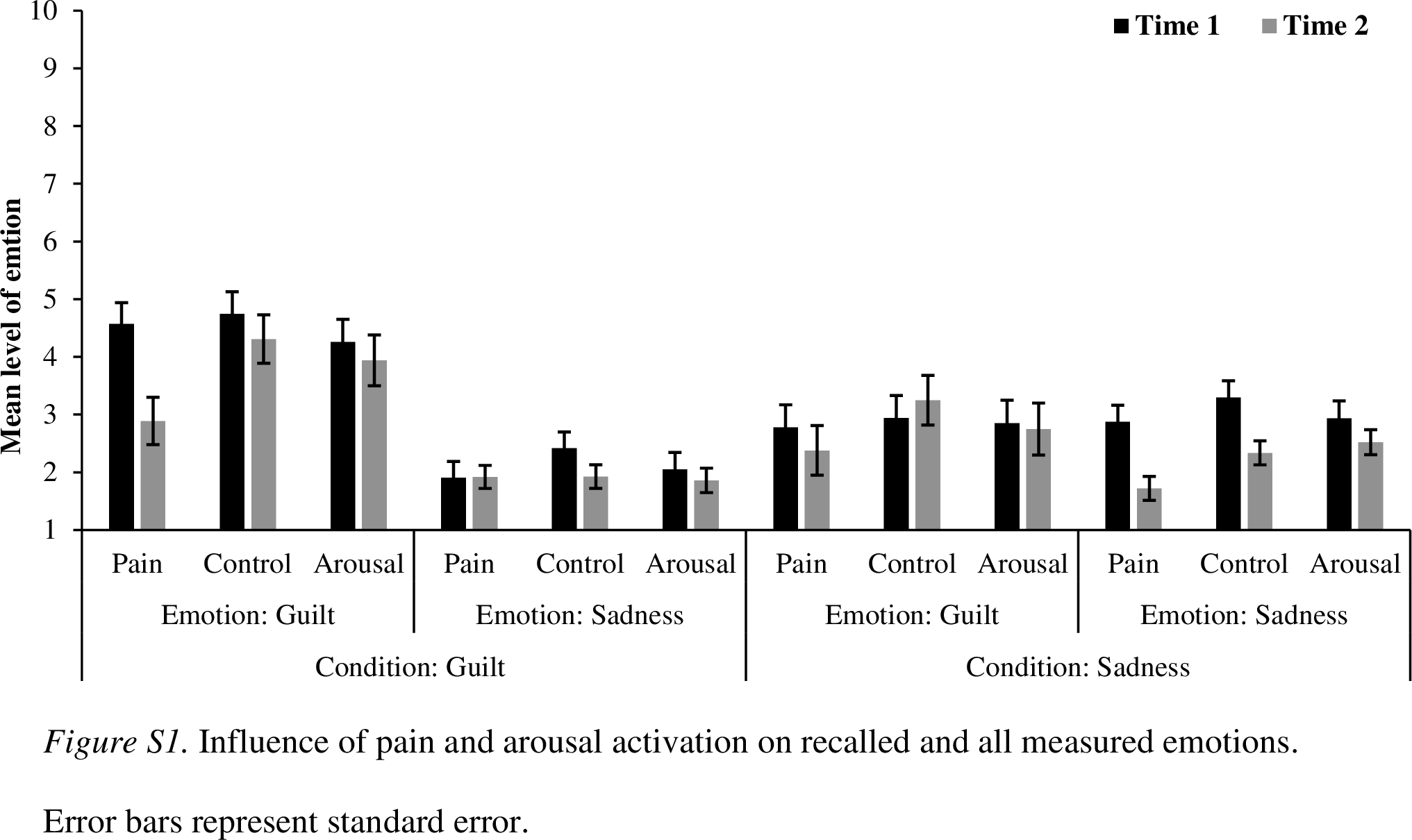


As presented in Table 1, our hypotheses confirmed, and we replicated the results from Study 1. In the pain condition, guilt decreased from Time 1 to Time 2 (*M* = 4.57, *SD* = 1.79 vs. *M* = 2.89, *SD* = 2.39) but only after the pain activation movie, *t*(27) = 3.01, *p* = .006, *d* = 0.67, 95% CI [0.13, 1.21]. In the control group and after the excitement activation movie, guilt remained at the same level (*p* = .574 and *p* = .582, respectively). For the sadness condition, sadness also decreased after the pain activation movie, *t*(25) = 3.54, *p* = .002, *d* = 0.53, 95% CI [0.02, 1.08], and in the control group, *t*(25) = 2.36, *p* = .026, *d* = 0.43, 95% CI [0.12, 0.98], but not in the excitement activation movie group (*p* = .367).


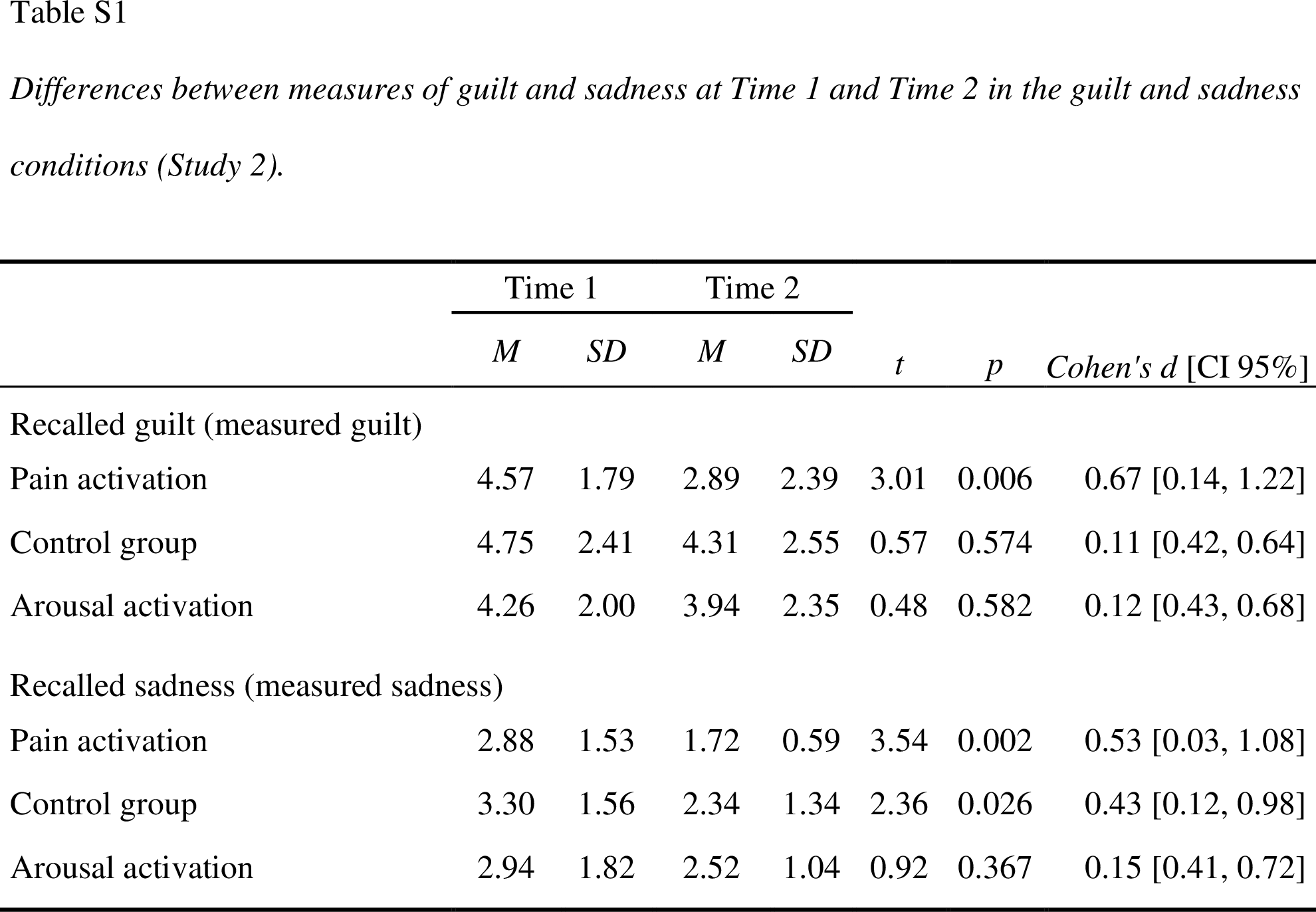


**Study 3**

In study 3, we additionally measured how painful the blood collection was according to the participants’ opinion and how much they identified with the protagonist. Participants were asked to report on 5-point scale their judgment from 1 = *not at all* to 4 = *extremely*. T-Test analysis showed no differences between the conditions for both pain perception, *t(*58) = .22, *p* = .825, and identification with the actor, *t(*58) = .40, *p* = .692.

**Full analyses**

**Guilt and shame*.*** We analyzed the data in a 2 (Condition: Guilt vs. Shame) x 2 (Measured Emotion: Guilt vs. Shame) x 2 (Time of the Measure: Time 1 vs. Time 2) mixed model of analysis of variance with the first factor between-group and two remaining within-group. The main effect of time was strong and significant, *F*(1, 58) = 61.00, *p* < .001, η_p_² *=* .51. At Time 2, participants felt less guilt and shame (*M* = 26.67, *SE* = 2.65) than at Time 1 (*M* = 50.10, *SE* = 3.47). The interaction between measured emotion and time of measure was also significant, *F*(1, 58) = 17.82, *p* < .001, η_p_^2^ *=* .24, and showed that shame decreased slightly from Time 1 (*M* = 47.93, *SD* = 43.37) to Time 2, (*M* = 34.77, *SD* = 34.67), *t*(59) = 3.38, *p* = .001, *d* = 0.34, 95% CI [0.18, 0.84], whereas guilt decreased two-fold: Time 1 (*M* = 52.27, *SD* = 30.39) versus Time 2 (*M* = 18.57, *SD* = 21.98); *t*(56) = 8.42, *p* < .001, *d* = 1.27, 95% CI (0.68, 1.83). The interaction between condition and measured emotion was also significant, *F*(1, 58) = 14.08, *p* < .001, η_p_^2^ *=* .20, and indicated that shame was stronger (*M* = 52.80, *SE* = 5.20) in the shame condition in comparison to the guilt condition (*M* = 29.90, *SE* = 5.20), but guilt was strong in both conditions: guilt condition (*M* = 37.95, *SE* = 4.01) and shame condition (*M* = 32.88, *SE* = 4.01). The last significant interaction was interaction between condition and time of measure, *F*(1, 58) = 6.06, *p* = .017, η_p_^2^ *=* 0.10. In the guilt condition, guilt and shame strongly decreased from Time 1 to Time 2 (*M* = 49.33, *SE* = 4.91 vs. *M* = 18.52, *SE* = 3.75), whereas in the shame condition, both emotions decreased slightly (Time 1: *M* = 50.87, *SE* = 4.91 vs. Time 2: *M* = 34.82, *SE* = 3.75). The interaction between condition, measured emotion and time of measure was nonsignificant, *F*(1, 58) = .10, *p* = .749. The summary of the analysis is presented in Figure S2.


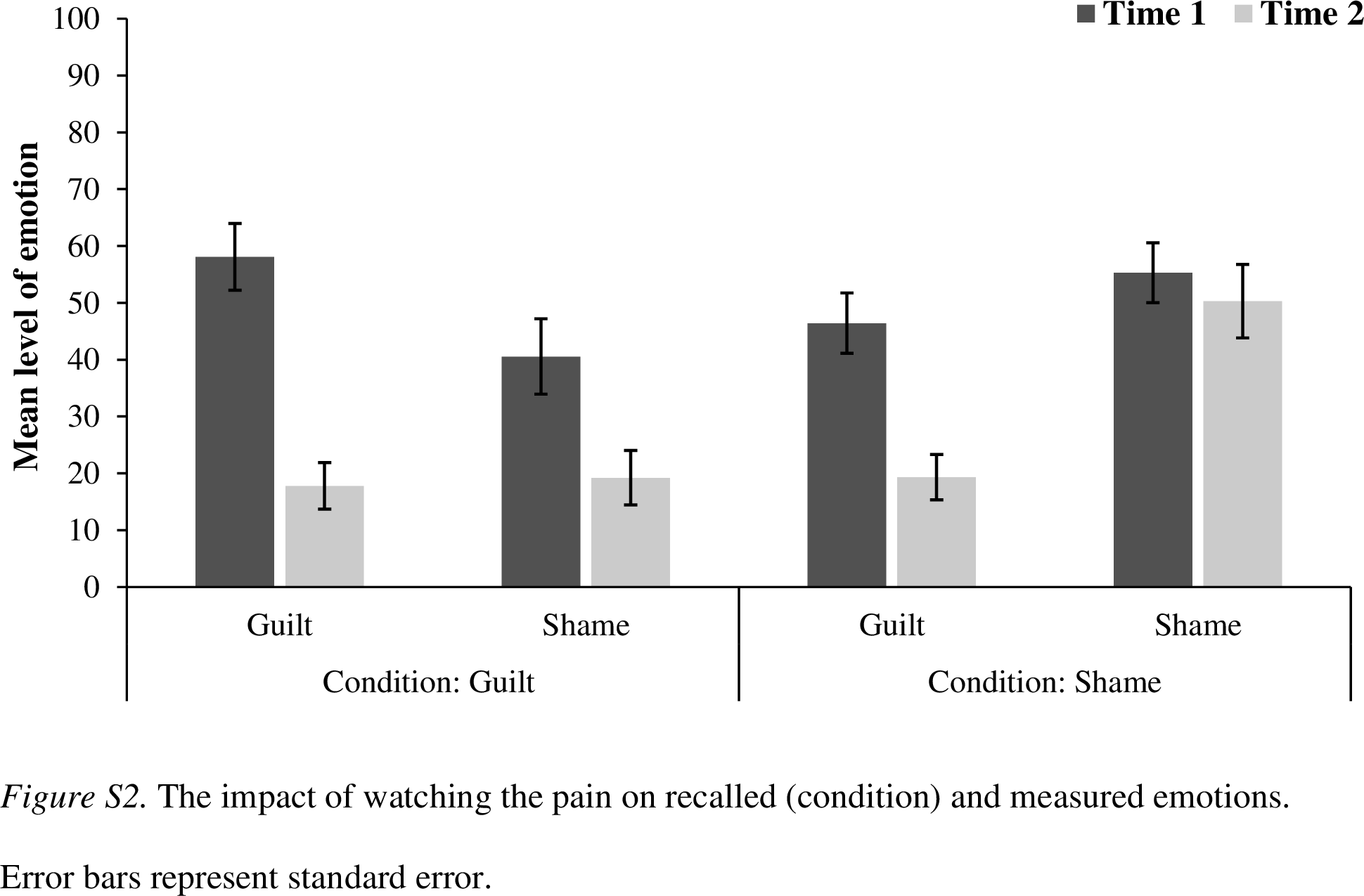


We applied a simple effect analysis to evaluate the hypothesis that only guilt would be affected by pain manipulation. As expected in the guilt condition, guilt decreased significantly from Time 1 to Time 2 (*M* = 58.10, *SD* = 32.13 vs. *M* = 17.89, *SD* = 22.49), *t*(29) = 7.17, *p < .*001. More importantly, we observed that shame in the shame condition remained at the same level at Time 2 (*p* = .362). When shame was triggered as a side effect of the guilt condition, we did observe a decrease after watching another person in pain, but the magnitude of the change was two times smaller than that for guilt. Interestingly, when guilt was triggered as a side effect of the shame condition, we found a decrease in guilt like in the guilt condition. Table S2 presents a summary of all simple effects.


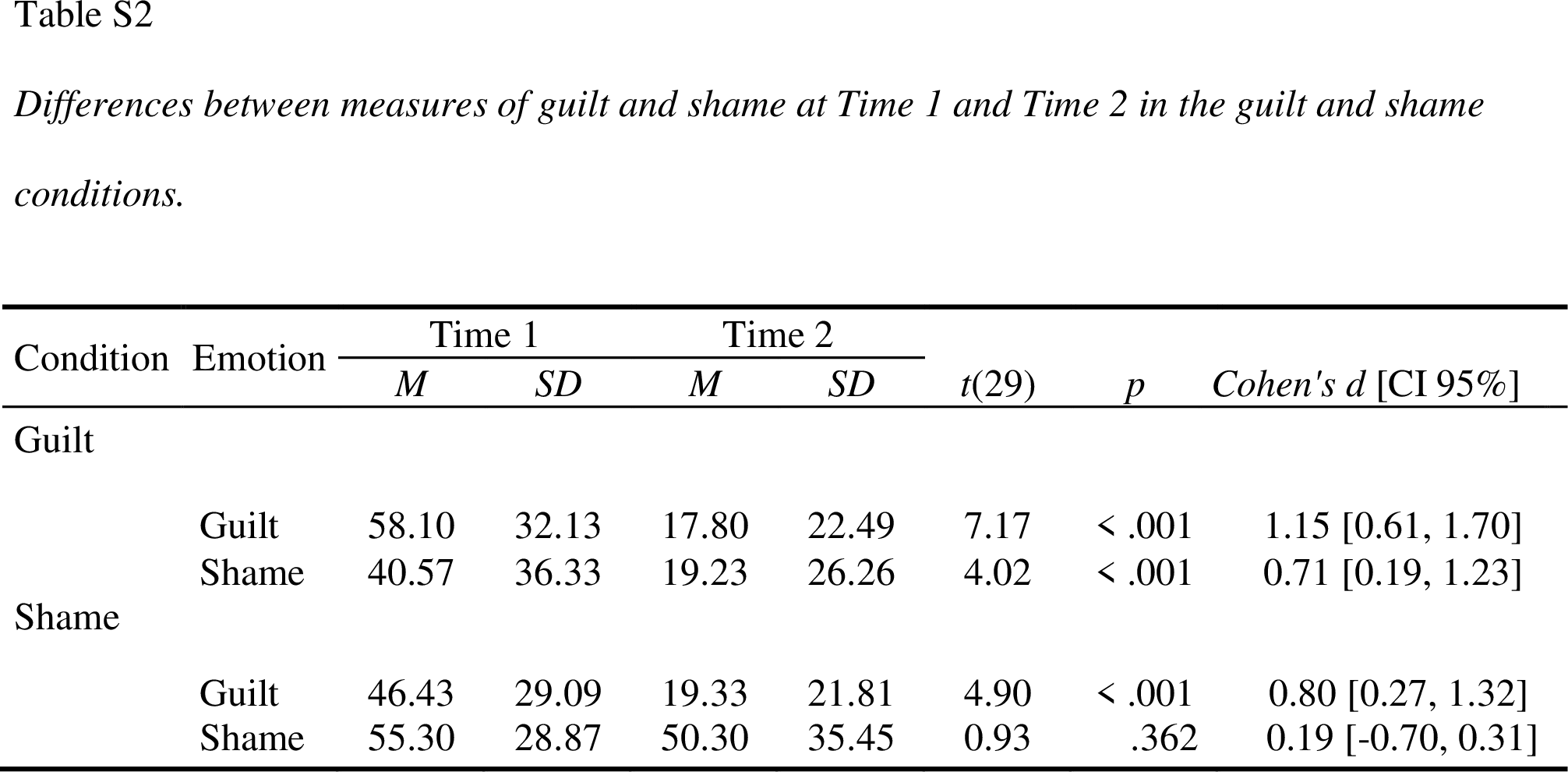

Supplement: S1 File — (DOCX) [file pone.0244429.s001.docx]
