# Supplementary material for: Pain(less) cleansing: Watching other people in pain reduces guilt and sadness but not shame
Source: PLoS One. 2020 Dec 30;15(12):e0244429. doi: 10.1371/journal.pone.0244429 (PMC7773247; doi:10.1371/journal.pone.0244429)
Supplement: Attachment — Submitted filename: PONE-D-20-01585 - Peer Review.docx [file pone.0244429.s002.docx]

The authors did an excellent job in their revisions and at addressing many of the issues with their original submission. Overall, the narrative and clarity of this manuscript have been substantially improved. However, there are still several issues that should be addressed:

**Introduction**

- I appreciate the authors’ attempts to clarify links between physical and social pain by adding the “Social Pain” section. However, one issue with linking this to guilt is that social pain is actually more closely linked to shame. Shame is thought to act as a warning signal that our need to belong is threatened and is activated in response to social exclusion. This should be noted in this section, and how this might impact the inferences made about links with shame vs. guilt in study 3.
- The sentence discussing participants freezing their hand and inhibition (end of pg. 5, start of pg. 6) is still unclear.
- Participants needing to select “agree” in Sona to proceed is a form of written consent. This should be clarified accordingly.

**Study 1**

- How many people were assigned to each condition?
- Specify that the grocery store condition is the control condition.
- In the first paragraph on page 9, it states that the other person is presumably a doctor. Is this unknown or is this meant to indicate that the participants are likely to presume it is a doctor?
- The last sentence on page 9 states “(any of the participants guessed the true aim of the study).” Should this say “none” instead of “any”?
- The fact that similar levels of sadness and guilt were observed in both conditions suggests an issue with the validity of your manipulations and should be noted in the discussion.

**Study 2**

- It is still unclear what is meant by “misattributing unrelated excitation”.
- Specify whether participants were randomly assigned and how many participants were assigned to each group.

**Study 3**

- On page 15, it is noted that shame is related to competitive behaviour, which is somewhat misleading.
- There is a discrepancy in the reasoning behind why guilt but not shame would be reduced in the study. In the Social Exclusion section in the introduction, you discuss activation of the ACC in response to social exclusion and that similar patterns are found both when we witness and when we experience social pain. This is used as a possible explanation for findings linking guilt reduction and contextual factors such as social exclusion. However, in the introduction section for study 3, it discusses how ACC activation is associated with shame but not guilt, and the different neural activation is used to justify why shame is hypothesized to remain the same in the study.
- In the second paragraph on page 15, it is noted that shame is elicited more often by other people, but then a few sentences later it is noted that guilt arises from interpersonal transactions.
- In the last paragraph of the introduction to study 3 (pg. 16), the hypothesized outcomes are discussed in terms of their relation with the ACC (e.g., “Therefore, we assume that on the one hand shame induction will recruit the ACC…”). This suggests the study will measure ACC activation, which it did not. Thus, these hypotheses should be reworded.
- Specify if participants were randomly assigned and how many participants were assigned to each group.
- The statement that the results of study 3 suggest shame my have a weaker association with self-punishment (pg. 18) does not really fit with this study considering the focus was on vicarious pain.

**General Discussion**

- The first sentence of the discussion (pg. 18) states the studies aimed to look at associations between guilt and self-punishment, however, as previously noted, this was not assessed in these studies.
- It is unclear how observing pain could have increased activation of the ACC (pg.20). If this were the case, would we not expect shame levels to also increase?

**Minor points:**

- Although substantially improved, there are still several spelling and grammatical errors throughout. The manuscript would benefit from a careful proofreading.
- Pg. 4 “…and in addition, such emotions as sadness and shame.” should be reworded
- Pg. 7, remove the sentence “In this article, we report all measures, all manipulations, and any data exclusions.”
- The verb tense in the description of guilt and sadness (pg. 9) is inconsistent
