# Supplementary material for: Pain(less) cleansing: Watching other people in pain reduces guilt and sadness but not shame
Source: PLoS One. 2020 Dec 30;15(12):e0244429. doi: 10.1371/journal.pone.0244429 (PMC7773247; doi:10.1371/journal.pone.0244429)
Supplement: Attachment — Submitted filename: Response to reviewers Minor REVISION.docx [file pone.0244429.s003.docx]

**RESPONSES TO ISSUES RAISED BY REVIEWER 1:**

**Introduction**

- **I appreciate the authors’ attempts to clarify links between physical and social pain by adding the “Social Pain” section. However, one issue with linking this to guilt is that social pain is actually more closely linked to shame. Shame is thought to act as a warning signal that our need to belong is threatened and is activated in response to social exclusion. This should be noted in this section, and how this might impact the inferences made about links with shame vs. guilt in study 3.**

We much appreciate this suggestion. However, we believe that evidence showing the link between shame and social pain fits better the section regarding Study 3 since, in this section, we want to underline the differences between shame and guilt. Moreover, the “Social Pain” section aimed to show evidence that people may feel pain without experiencing physical pain instead of presenting evidence for the link between social pain and guilt. Since our manipulation of pain observation was not related to social exclusion and therefore, to social pain, we do feel that it makes no sense to write about the link between guilt and social pain in the “Social Pain” section. Therefore, we removed this part from the “Social Pain” section:

*Therefore, on the one hand, it is possible that the guilt-reducing effect of pain found in the past research (Bastian et al., 2011; Inbar et al., 2013) may be triggered by contextual factors (e.g., social exlusion). On the other hand, the guilt-reducing effect of pain may by triggered when people observe another’s pain.*

Nevertheless, we recognise the importance of the link between social pain and shame in light of Study 3. Thus, on p. 15-16, we write:

*Research concerning the neural activity of brain regions shows another difference between shame and guilt. Experience of shame is associated with increased activation in the ACC (Krach et al., 2011; Michl, 2014), the same brain region, which is active when people experience physical pain, social pain and when they observe pain in others. Furthermore, research has suggested a specific link between social pain and shame, because social exclusion increases shame and this link is mediated by feelings of being devalued (Robertson, Sznycer, Delton, Tooby & Cosmides, 2018). These results correspond with cross-cultural research which has shown that shame is strongly associated with tracking the magnitude of the devaluative threat in others (Sznycer, Tooby, Cosmides, Porat, Shalvi & Halperin, 2016) or evidence that, people who have experienced shame prefer to be together with others over being alone (Hooge, Breugelmans, Wagemans & Zeelenberg, 2018).*

- **The sentence discussing participants freezing their hand and inhibition (end of pg. 5, start of pg. 6) is still unclear.**

Thank you for this comment. We tried to make the sentence clearer and now, on p. 5 we write:

*For example, participants’ hand muscles potentials are reduced when they see another’s hand being hurt, but such inhibition in the sensory-discriminative component of pain was not found when they saw a needle penetrating a tomato or cotton swab moving over the hand. This muscle inhibition suggests activation of a pain resonance system that extracts essential sensory aspects of the other person’s painful experience (Avenanti, Bueti, Galati, & Aglioti, 2005).*

- **Participants needing to select “agree” in Sona to proceed is a form of written consent. This should be clarified accordingly.**

Thank you. We clarified this and now, on p. 7 we write:

*We obtained the written consent from participants via Sona platform. Participants proceeded to the study only when they agreed to participate in the study after reading the description of the study, time involved, and data policy.*

**Study 1**

- **How many people were assigned to each condition?**

In each condition we had 20 participants. We have added this information o p. 8.

- **Specify that the grocery store condition is the control condition.**

Corrected on p. 8.

- **In the first paragraph on page 9, it states that the other person is presumably a doctor. Is this unknown or is this meant to indicate that the participants are likely to presume it is a doctor?**

This information is unknown. We write on p. 8:

*In the movie, participants observed the forearm of an unknown person and another person (presumably a doctor, however this information was unknown) trying to insert a needle into the vein of the examined person.*

- **The last sentence on page 9 states “(any of the participants guessed the true aim of the study).” Should this say “none” instead of “any”?**

Corrected on p. 9.

- **The fact that similar levels of sadness and guilt were observed in both conditions suggests an issue with the validity of your manipulations and should be noted in the discussion.**

We appreciate this suggestion. Now, o p. 11, we write:

*On the one hand, this finding is likely because, in addition to inducing sadness in this condition, we also elicited guilt. On the other hand, similar levels of sadness and guilt observed in both conditions might suggest some issues with the validity of manipulation used in Study 1.*

**Study 2**

- **It is still unclear what is meant by “misattributing unrelated excitation”.**

We clarified this, and now, on p. 11, we write:

*Specifically, we investigated an idea that unrelated to the pain observation arousal evoked by intensely exciting stimuli instead of a painful one, might later reduce guilt.*

- **Specify whether participants were randomly assigned and how many participants were assigned to each group**.

Corrected. Now, on p. 12, we write:

*Based on a random assignment, we asked participants to recall a time when they felt guilty or sad, and then we asked them to either watch the painful movie clip from Study 1, the exciting movie clip or the neutral movie clip (N ~ 26 for each group).*

**Study 3**

- **On page 15, it is noted that shame is related to competitive behaviour, which is somewhat misleading.**

Thank you for this suggestion. We removed the phrase “competitive behaviour” and now we write on p. 15:

*Moreover, guilt evolved from a need to avoid harming others (Tangney & Dearing, 2002), while in contrast, shame is a self-focused emotion that is related to needs to prove oneself as acceptable or desirable to others (Gilbert, 2003).*

- **There is a discrepancy in the reasoning behind why guilt but not shame would be reduced in the study. In the Social Exclusion section in the introduction, you discuss activation of the ACC in response to social exclusion and that similar patterns are found both when we witness and when we experience social pain. This is used as a possible explanation for findings linking guilt reduction and contextual factors such as social exclusion. However, in the introduction section for study 3, it discusses how ACC activation is associated with shame but not guilt, and the different neural activation is used to justify why shame is hypothesized to remain the same in the study.**

We much appreciate this comment. After carful read, we notice that the part of the paragraph which mentioned contextual factors was confusing. Therefore, we removed it and now we write on p. 5:

*Overall, the evidence we reviewed suggests that people may feel a pain event without directly experiencing psychical pain. In fact, we have ample evidence showing that the vicarious experience of pain can be generated when people observe another’s pain. Therefore, peoples’ physiological response might be similar to, but qualitatively different from, physical pain, or people might report feeling pain themselves when seeing others in pain (Avenanti, Pauello, Bufalari, & Aglioti, 2006; Jackson, Rainville, & Decety, 2006; Lamm, Nusbaum, Meltzoff, & Decety, 2007; Osborn & Derbyshire, 2010).*

- **In the second paragraph on page 15, it is noted that shame is elicited more often by other people, but then a few sentences later it is noted that guilt arises from interpersonal transactions.**

Thank you for this comment. We clarified the paragraph about the differences between guilt and shame and now we write on p. 15:

*Cross-cultural studies have found that participants reported that their shame experiences were elicited more often by other people or external sources than by guilt experiences (Wallbott & Scherer, 1995; see also Fontaine, Luyten, de Boeck, & Corveleyn, 2006). Further, research has demonstrated that when people experience shame, they tend to focus on themselves, but when they experience guilt, they tend to focus on their behaviour (Niedenthal, Tangney, & Gavanski, 1994). Finally, shame arises when adverse events are attributed to one’s stable, global self while guilt arises when adverse events are attributed to unstable, specific aspects of the self (Tracy & Robins, 2004). Moreover, guilt evolved from a need to avoid harming others (Tangney & Dearing, 2002), while in contrast, shame is a self-focused emotion that is related to needs to prove oneself as acceptable or desirable to others (Gilbert, 2003).*

- **In the last paragraph of the introduction to study 3 (pg. 16), the hypothesized outcomes are discussed in terms of their relation with the ACC (e.g., “Therefore, we assume that on the one hand shame induction will recruit the ACC…”). This suggests the study will measure ACC activation, which it did not. Thus, these hypotheses should be reworded.**

Following the reviewer’s suggestion, we reworded this part. No, we write on p. 16:

*Overall, presented evidence suggests that experiences of shame, physical pain, and social pain share the same neural basis because they are all associated with increased activation in the ACC (Eisenbeger, 2003; Krach et al., 2011; Michl, 2014; Rainville et al., 1997; Sawamoto et al., 2000). Therefore, it is plausible that shame levels would remain intact after pain observation because the same brain regions process both shame and observation of another person in pain (e.g., ACC). On the other hand, discovered in Study 1 and Study 2 the guilt-reducing effect of pain observation may occur because different brain regions process guilt (e.g., OFC) and these brain regions are not involved in pain processing.*

- **Specify if participants were randomly assigned and how many participants were assigned to each group.**

Corrected. Now we write on p. 17:

*Thus, based on a random assignment, we asked participants to recall and describe as precisely as possible a particular event from their life when they either: 1) through their own fault, acted against their own rules, and they caused harm to another person by their fault (the guilt condition, N = 30) or 2) their reputation was damaged, and they could not to change the negative impression that they caused (the shame condition, N = 30).*

- **The statement that the results of study 3 suggest shame my have a weaker association with self-punishment (pg. 18) does not really fit with this study considering the focus was on vicarious pain.**

Thank you. We changed self-punishment with the vicarious pain experience. No, we write on p. 18:

*The results of Study 3 corroborated findings from Studies 1 and 2 and provide additional evidence that watching other people in pain reduces feelings of guilt. Additionally, we found that shame remained intact after participants watched another person in pain. This pattern of results suggests that watching other people in pain influences levels of guilt and shame differently when a target emotion is recalled. The results of Study 3 also suggest that shame, as opposed to guilt, might show weaker associations with the vicarious pain experience.*

**General Discussion**

- **The first sentence of the discussion (pg. 18) states the studies aimed to look at associations between guilt and self-punishment, however, as previously noted, this was not assessed in these studies.**

Thank you. We corrected this sentence and now we write on p. 19:

*In this paper, we sought to contribute to the scarce research on the guilt-reducing effect of pain (Bastian et al., 2011; Inbar et al., 2013; van Bunderen & Bastian, 2014) by finding evidence for a specific association between guilt and mere observation of others in pain.*

- **It is unclear how observing pain could have increased activation of the ACC (pg.20). If this were the case, would we not expect shame levels to also increase?**

Thank you. We removed the word “increase” and now we write on p. 20:

*Relying on these premises, on the one hand, we assumed that watching other people in pain would recruit the ACC, which past studies have proposed to be an important cortical brain region responsible for pain perception (Casey, Minoshima, Morrow, & Koeppe, 1996; Becerra et al., 1999; Talbot et al., 1991). On the other hand, shame also recruits the ACC (Michl, 2014), therefore, we assumed that shame induction would recruit the ACC and further pain observation sustained this activation. Obviously, these results should be treated with great caution because we did not test activation of the ACC and other brain regions directly involved in guilt and shame processing.*

**Minor points:**

- **Although substantially improved, there are still several spelling and grammatical errors throughout. The manuscript would benefit from a careful proofreading.**

We believe that some spelling issues are because the text was proofread according to British English rules. However, we again carefully proofread the new version of the manuscript.

- **Pg. 4 “…and in addition, such emotions as sadness and shame.” should be reworded**

We reworded the sentence. Now, on p. 4 we write:

*Specifically, we aimed to examine if observing others’ pain impacts guilt. Furthermore, we tested whether observing others’ pain would affect sadness and shame as well.*

- **Pg. 7, remove the sentence “In this article, we report all measures, all manipulations, and any data exclusions.”**

We disagree with the reviewer that this sentence should be removed. This kind of statement is related to guidelines proposed by the open science movement since it improves the transparency of the paper and also states what kind of practices were used during the data analyses.

- **The verb tense in the description of guilt and sadness (pg. 9) is inconsistent**

Thank you. We corrected this issue. Now on p. 9 we write:

*By using a scale from 1 = very slightly or not at all to 5 = extremely, participants indicated to what extend they felt guilty and upset.*

**RESPONSES TO ISSUES RAISED BY REVIEWER 2:**

**Main points:
1.     One of the main methodological concerns is the sample size of each study. The authors provide post hoc power calculations, but it is unclear how they arrived at these numbers or what they mean. Please provide a more detailed account of the power that was achieved by the current sample per study. Please follow the steps outlined by Daniel Lakens here and explain how likely it was to observe a significant effect, given your sample, and given an expected or small effect size and report all parameters entered in G*Power: http://daniellakens.blogspot.com/2014/12/observed-power-and-what-to-do-if-your.html.
   a.     It is a bit unclear why the authors report f effect sizes when their main analyses are repeated measures ANOVAs**.

Based on the reviewer’s suggestion, we provide more information about the effects found in the present studies. Specifically, instead of post-hoc power calculations, we run sensitive power analysis for each study. For example, now we write that for Study 1, our sample size provides 0.80 power for the detection of an effect size of *f2* = .21. Also, we report f effect size because we run power analyses for interactions effects.

**2.     A conceptual issue relates to the idea that “mere observation of others in pain produces a full experience of pain in the observer” (p. 4). That statement seems hyperbolic and scientifically incorrect based on the research cited. It is true that observing someone else in pain activates similar neural regions than direct experience of pain, but it is also clear that the actual experience of observing someone else in pain and experiencing pain oneself is qualitatively quite different. For instance, no one would confuse whether they experienced pain themselves or they saw someone else experiencing pain. Moreover, the authors did not assess in any way what emotional reaction the participants had to the video of someone else in pain, how “painful” they experienced watching it, or how they were affected by it. In my mind, these are crucial variables to test the hypothesis the authors put forward. Could the authors comment?**

We much appreciate this comment. After a careful read of the manuscript, we decide to rewrite the parts of the manuscript regarding the vicarious pain experience and remove parts which were unclear as the one mentioned by the reviewer. Specifically, we added the section about the social pain o p 4. Moreover, now on p. 5-6, we write:

*From a neurological standpoint, pain is multidimensional and covers three broad components: the sensory-discriminative component (the intensity of pain and its bodily location), the affective component (the experience of the unpleasantness) and the cognitive component (de Vignemont & Jacob, 2012; Zaki, Wager, Singer, Keysers, & Gazzola, 2016). These components, due to the observation of another’s pain, might be activated and may produce an experience similar to the experience of physical pain. For example, participants’ hand muscles potentials are reduced when they see another’s hand being hurt, but such inhibition in the sensory-discriminative component of pain was not found when they saw a needle penetrating a tomato or cotton swab moving over the hand. This muscle inhibition suggests activation of a pain resonance system that extracts essential sensory aspects of the other person’s painful experience (Avenanti, Bueti, Galati, & Aglioti, 2005). A different study found activity in the primary somatosensory cortex of participants who observed the needle penetrating the hand (Bufalari, Aprile, Avenanti, Di Russo, & Aglioti, 2007). This evidence suggests that observing which part of the body was hurt and how intensively it was hurt might trigger vicarious pain in the observer via activation of the sensory-discriminative component of pain.*

*Vicarious pain experience can be triggered by the affective component of pain, too. Research has demonstrated that the affective component of pain was activated when participants were viewing facial expressions of pain (Botvinick et al., 2005). Similar activation of the affective component of pain was found when participants observed a signal indicating that their loved one receives pain stimulus (Singer et al., 2004). Moreover, we have evidence suggesting that the activation of affective component of pain can be modulated by a wide range of personal and contextual factors, such as the moral behaviour of the person in pain (Singer et al., 2006), his or her medical expertise (Cheng et al., 2007) or pain resulting from medical treatment (Lamm, Batson, & Decety, 2007).*

*Cumulatively, research on social and vicarious pain experience suggests that people might report feeling the pain without being physically hurt. Because past evidence has suggested that after the painful experience participants’ levels of guilt decreased after the painful experience (Bastian et al., 2011; Inbar et al., 2013), we propose that observing another’s pain might generate in the observer a superficially similar but qualitatively different kind of painful experience and therefore generate the guilt-reducing effect of pain.*

**a.     Similarly, the authors keep speaking of “experiencing vicarious pain” without assessing it and is therefore speculative. I would prefer if the authors change this to “observing someone else in pain” or “vicarious pain perception” throughout the whole manuscript and in the title as this more clearly describes the manipulation which was employed.**

We much appreciate the reviewer’s suggestions. In the revised version of the manuscript, we change term the “vicarious pain experience”, and instead we use term “pain observation”, “watching other people in pain” or “observe another’s pain” were possible. However, we believe that in some parts of the manuscript we should stick to the term “vicarious pain experience” or “vicarious experience of pain” because these are terms used in the literature describing effects of the pain observation in others.

**3.     There are several grammatical and language errors in the text, and the manuscript would profit from professional academic proofreading.**

The manuscript was proofread by professional service with the help of two British English native speakers.

**Minor points:

1.     P. 2 “pain observation affected participants’ feelings of guilt” Please state the direction of the effect**

Now we write on p. 2:

*Finally, in Study 3 (N = 60), pain observation lowered participants’ feelings of guilt but not their feelings of shame.*

**2.     P. 6 “shame and pain experience share the same neural underpinnings” What does that mean and why does that mean it would not be affected?**

This statement is not present anymore in the revised version of the manuscript. However, we explain the difference in the neural activity of brain regions regarding shame, guilt and pain on p. 15 and 16. Also, we added a whole new section about social pain on p. 4

**3.     P. 7 “would be observed after watching an exciting and neutral movie clip” Why not use an arousing negative clip as a comparison? It would make the claim stronger that the guilt reduction is in response to pain alone.**

We much appreciate this suggestion. At the time of planning experiments, we had access to arousing clip only. Also, we did not think of that the arousing negative clip could be a better option for our claim. Nevertheless, we agree with the reviewer’s suggestion that use of arousing negative clip would make our claim stronger.

**4.     P. 8 “asked to write about the last time” How long were the participants required to write? Did all participants write for the required time?**

We did not give participants any time restrictions regarding the recall task. Therefore, we did not measure how long participants wrote their memories.

**5.     P. 8 Please include a paragraph on the actual design of the study. All studies employed (at least in part) a between-subjects design yet this is not explicitly stated anywhere. Also, the n of each group is not stated or how participants were randomized. Lastly, please refer to between-subject levels as “groups” rather than “conditions” throughout the manuscript.**

Following the reviewer’s suggestion, we added information about the between-subjects design in the results sections for all three studies. We also changed groups for conditions.

**6.     P. 9 “they were told to put their stories in an envelope” What was the cover story and what did the participants think would happen with the stories? Were they told that they were kept confidential?**

The cover story for the experiment was that we are interested in how self-reflection can help people cope with negative life events. There was no cover story for the envelope. We wanted to assure participants that their stories are safe, anonymous and confidential even though we did not say that explicitly.

**7.     P. 9 “were measured with two items” Please state the exact wording, scale and labels of the items used.**

We added exact wording, scale and labels. On p. 9 we write:

*Guilt and sadness were measured with two items from the 20-item Positive and Negative Affect Schedule (PANAS; Watson, Clark & Tellegen, 1988). By using a scale from 1 = very slightly or not at all to 5 = extremely, participants indicated to what extend they felt guilty and upset.*

**8.     P. 9 Please include a separate paragraph one the exact statistical analyses that were carried out per study, including the dependent and independent variables, their levels and the used alpha-level.**

Information about the detailed statistical analyses that were carried out per study is provided in the results sections.

**9.     P. 10 “we conducted an ANOVA” Please specify what type of ANOVA (in this case a repeated measures ANOVA)**

On p. 19 we write:

*To test the hypothesis that guilt may be reduced by observing another’s pain, we conducted a mixed ANOVA analysis in a 3 (Condition: Guilt vs. Sadness vs. Control) x 2 (Guilt: Time 1 vs. Time 2) design with the first factor as between-subjects and the second factor as within-subjects.*

**10.     P. 11 “the participants’ level of sadness decreased after the pain manipulation” This is a very surprising finding and definitely requires further elaboration in the discussion. Earlier research shows that watching someone else in pain elicits unpleasant emotions and it is likely that one would feel sad for someone else in pain (especially with high empathy levels). So how do you explain a reduction in sadness when observing someone else in pain?**

As we stated in the paper, we recognise that the results we found are challenging and puzzling. First on p. 11 we write:

*In contrast to past evidence that sadness does not motivate people to seek self-punishment (Inbar et al., 2013), we found that observing another’s pain affected participants’ feelings of sadness. This result is somewhat unexpected and challenging to explain. On the one hand, unlike past research (Inbar et al., 2013), participants in our study could not decide how much pain they wanted to inflict on themselves. Instead, they had to observe another person in pain. Thus, this difference might explain why the participant’s sadness was affected by pain observation manipulation. On the other hand, sadness might fade away with time or because of the intense arousal triggered when participants were watching another person in pain. We sought to examine this alternative explanation in Study 2.*

Then, on p. 19-20, we write:

*The original contribution of these studies to the current literature is threefold: First, we contribute to the past research on the guilt-reducing effect of pain by presenting a novel approach to studying how pain observation affects guilt (Bastian et al., 2011; Inbar et al., 2013). Second, in Studies 1 and 2, we found evidence that sadness also decreased after observing another’s pain. Research by Inbar et al. (2013) showed that guilt motivates people to self-punishment more strongly than sadness. In the authors’ study, in all three conditions (guilt, sadness, and control), participants inflicted electroshocks on themselves, but in the guilt condition, participants gave themselves stronger shocks than in the other two conditions. The analysis of guilt at Time 1 and Time 2 showed that stronger shocks were associated with more alleviation of feelings of guilt (Inbar et al., 2013). Unfortunately, there is no information about levels of sadness and the impact that electric shocks had on this emotion.*

*It is at least plausible that sadness is also affected by painful experience but in a weaker way than guilt. We found some support for this hypothesis in Study 1. Both guilt and sadness decreased after pain observation, but the size of the effect was more than twice the size for guilt (d = 1.02) than for sadness (d = .45). However, in Study 2, sadness decreased in both the pain and the control groups, thereby possibly implying that sadness wears off over time. More research about sadness in the context of self-punishment and the experience of pain observation is needed.*

**11.     P. 11 In general, there are quite a few post-hoc comparisons. Did the authors employ any post-hoc corrections such as Holm-Bonferroni to correct for multiple testing? This is especially relevant since the studies are likely underpowered.**

For the post-hoc comparisons, we used Bonferroni or Tamhane test.

**12.     P. 11 “apart from inducing sadness in this condition, we also elicited guilt” Please elaborate. What do you mean?**

We corrected the wording. Now we write on p.11:

*On the one hand, this finding is likely because, in addition to inducing sadness in this condition, we also induced guilt.*

**13.     P. 11 “(Bastian et al., 2011 (…)” bracket missing**

Corrected.

**14.     P. 13 “We changed the way guilt and sadness was measured” Why did the authors choose to change their measurements throughout the studies? If they want to present it as a replication of Study 1 it seems counterintuitive to change the methodology. Please provide arguments for doing so.**

We agree that probably it would be better to use the same measure of guilt and sadness. However, because we did not change the way of how we induced these two emotions, we decided to change the way of how we measured them. Therefore, we wanted to increase the external validity of Study 2.

**15.     P. 13 “was measured with four items” How were these items combined in a single outcome variable? Since this is an entirely new measure, please provide psychometric properties (e.g. Cronbach’s Alpha).**

On p. 12-13 we write:

***Guilt*** *was measured with two items: “guilt” and “remorse”. Participants indicated to what extent they feel each emotion by using a 10-point scale from 1 = I do not feel it at all to 10 = I feel it extremely strongly (Time 1, α = 0.50, M = 3.72, SD = 2.13; Time 2, α = 0.80, M = 3.26, SD = 2.27).*

***Sadness*** *was measured with four items: “depression”, “sadness”, “misery”, and “breakdown”. Participants rated how they feel each emotion by using a 10-point scale from 1 = I do not feel it at all to 10 = I feel it extremely strongly (Time 1, α = 0.71, M = 2.57, SD = 1.53; Time 2, α = 0.46, M = 2.04, SD = 1.08).*

**16.     P. 14 Did you assess perceived pain intensity or empathy levels at any point?**

Yes, we did. The analyses are provided in the Supplement:

*In study 1, we additionally measured how painful the blood collection was according to the participants’ opinion and how much they identified with the protagonist. Participants were asked to report on 5-point scale their judgment from 1 = not at all to 4 = extremely. One-way ANOVA showed no differences between all three conditions for both pain perception, F(2,57) = 1.38, p = .259, and identification with the actor, F(2,57) = 1.03, p = .365.*

*In study 2, we additionally measured how painful the blood collection was according to the participants’ opinion and how much they identified with the protagonist. Participants were asked to report on 5-point scale their judgment from 1 = not at all to 5 = extremely. One-way ANOVA showed no differences between all conditions for both pain perception, F(2,150) = .32, p = .730, and identification with the actor, F(2,150) = .67, p = .513.*

*In study 3, we additionally measured how painful the blood collection was according to the participants’ opinion and how much they identified with the protagonist. Participants were asked to report on 5-point scale their judgment from 1 = not at all to 4 = extremely. T-Test analysis showed no differences between the conditions for both pain perception, t(58) = .22, p = .825, and identification with the actor, t(58) = .40, p = .692.*

**17.     P. 16 “past studies proved” Please avoid the word “proved” in scientific writing.**

Thank you for this suggestion. We removed word “proved” from the revised version of the manuscript.

**18.     P. 18 “Finally, using Bayesian interference” The authors introduce a whole new set of statistical analyses here. Please be consistent throughout your studies in the analyses employed and if not, provide a clear rationale for changing method of analysis.**

After considering the reviewer's suggestion, we decide to remove Bayesian interference analysis from the manuscript.

**19.     P. 22 “might help people deal with aversive feelings of guilt.” The notion that people watch violent shows because of feelings of guilt is highly speculative and far-fetched. I would reduce this last paragraph. Instead, I would be interested in practical or clinical implications of this research as these are currently missing in the discussion and introduction.**

We much appreciate this suggestion. We removed from the manuscript the part about violence and instead added a section “Conclusion”. Now on p. 21 we write:

*By systematically examining whether watching other people in pain reduces feelings of guilt, this research provides additional support for the theories that argue that guilt leads to a need for suffering (Bastian et al., 2011; Baumeister et al., 1994; Freud, 1924/1953; Inbar et al., 2013). The results of this research suggest that the guilt-reducing effect of pain occurs even without the experience of physical pain. Therefore, we conclude that merely observing other people in pain is sufficient to soothe our feelings of guilt.*
